# Particle filter-based parameter estimation algorithm for prognostic risk assessment of progression in non-small cell lung cancer

**DOI:** 10.1186/s12911-023-02373-3

**Published:** 2023-12-20

**Authors:** Shi Shang, Junyi Yuan, Changqing Pan, Sufen Wang, Xuemin Tu, Xingxing Cen, Linhui Mi, Xumin Hou

**Affiliations:** 1 Information Center, Shanghai Chest Hospital , School of Medicine, Shanghai Jiao Tong University, Shanghai, China; 2 Hospital’s Office, Shanghai Chest Hospital , School of Medicine, Shanghai Jiao Tong University, Shanghai, China; 3 Glorious Sun School of Business and Management, Donghua University, Shanghai, China; 4 Department of Mathematics, University of Kansas, Lawrence, KS USA

**Keywords:** NSCLC, Risk assessment model, Particle filtering, Parameter estimation

## Abstract

Non-small cell lung cancer (NSCLC) is a malignant tumor that threatens human life and health. The development of a new NSCLC risk assessment model based on electronic medical records has great potential for reducing the risk of cancer recurrence. In this process, machine learning is a powerful method for automatically extracting risk factors and indicating impact weights for NSCLC deaths. However, when the number of samples reaches a certain value, it is difficult for machine learning to improve the prediction accuracy, and it is also challenging to use the characteristic data of subsequent patients effectively. Therefore, this study aimed to build a postoperative survival risk assessment model for patients with NSCLC that updates the model parameters and improves model accuracy based on new patient data. The model perspective was a combination of particle filtering and parameter estimation. To demonstrate the feasibility and further evaluate the performance of our approach, we performed an empirical analysis experiment. The study showed that our method achieved an overall accuracy of 92% and a recall of 71% for deceased patients. Compared with traditional machine learning models, the accuracy of the model estimated by particle filter parameters has been improved by 2%, and the recall rate for dead patients has been improved by 11%. Additionally, this study outcome shows that this method can better utilize subsequent patients’ characteristic data, be more relevant to different patients, and help achieve precision medicine.

## Background

Lung cancer is one of the leading causes of death worldwide, and approximately 1.8 million people died of this disease in 2020 [1]. Radical resection is usually the first choice for non-advanced metastases in treating common types of lung cancer, particularly non-small cell lung cancer (NSCLC) [2, 3]. However, the postoperative recurrence rate of NSCLC is high, reaching approximately 34%, which seriously influences patient prognosis. There are clear differences in the prognosis of different stages of NSCLC. To reduce the damage caused by this disease, comprehensive treatment after surgery is a necessity, and after stage I–V NSCLC, the tumor, node, and metastasis (TNM) system is usually used to guide the specific program. Nevertheless, the TNM staging is generic, and other factors affecting the NSCLC prognosis, including differentiation type, vascular tumor thrombus, nerve invasion, and the number of lymph node dissections, are excluded [4–6]. Hence, it is essential to build a precise postoperative risk assessment model for NSCLC by considering other detailed factors to provide a reference for differentiated treatment and improved prognosis [7].

In exploring the NSCLC prognosis, traditional studies have mainly been based on retrospective analysis of large data samples from electronic medical records (EMR). Many studies have conducted risk factor exploration based on Kaplan–Meier one-way analysis of variance (ANOVA) or Cox regression multifactor ANOVA, which have achieved good results in summarizing influencing factors [8]. Similarly, EMR provides a good foundation for developing prognostic survival models for patients with NSCLC and adjuvant postoperative differential therapy [9, 10]. However, traditional methods only describe risk factors and cannot provide the weight of risk factors and patient survival rate under the influence of each factor, which has an adverse effect on the precise prognosis and treatment of NSCLC.

Currently, machine learning (ML) is a state-of-the-art method in the field of NSCLC prognostic risk assessment modeling. Many ML approaches, including logistic regression, artificial neural networks, decision trees, and SVM, have been established using EMR-extracted tumor marker data and have shown promising outcomes [11, 12]. However, ML methods frequently do not go through a single finite training process, requiring a steady stream of new training data to ensure the model’s predictive accuracy over time. If the risk evaluation model is not periodically retrained as the real-world variables evolve, the model’s accuracy will naturally decline over time, also known as data drift, concept drift, or model decay [13]. Traditional methods, including active detection, significantly improve the data drift problem by replicating part of the initial training data and adding unused data to form a new dataset for the manual training and updating of the model. However, these methods require drift detection and constant monitoring of the model performance [14], and the optimal correction period may have been missed when a decline in model capability is detected.

In the data assimilation process, both prediction and update processes involve the calculation of integration, and the Monte Carlo algorithm is often introduced to solve integration in practical problems. This algorithm converts the integral into its expected form by generating samples that obey the target distribution function and weighing the average to obtain the integral result. The Ensemble Kalman Filter (EnKF) is a classical data assimilation algorithm, which is a combination of ensemble forecasting and Kalman filtering methods from the mid-1990s. The algorithm is based on the Monte Carlo method to calculate the forecast error covariance of states, and the problem of difficulty in estimating and forecasting the background error covariance matrix in practical applications is solved by the idea of an ensemble. The EnKF is easy to implement and can be computed in parallel; however, filter scattering often occurs in practical applications, which shows that the analysis value will be closer to the background field as the assimilation time increases, and eventually, the observation data will be completely rejected.

A particle filter, also known as the sequential Monte Carlo filter, was developed based on the idea of sequential importance sampling filtering. The algorithm finds a set of random samples propagating in the state space to approximate the probability density function and replaces the integration operation with the sample mean to obtain the state minimum variance estimate. As the number of particles increases, the probability density function of the particles gradually approximates the probability density function of the state. Finally, the effect of optimal Bayesian estimation can be achieved.

In this study, we propose a novel particle filter-based NSCLC risk assessment ML method that is capable of continuous learning by incorporating new data streams from the production environment. First, structured data are extracted from the EMR, and dimensionality reduction is performed. The prognosis model of patients with NSCLC was then established using a logistic regression model. Next, the coefficients and intercepts of the model are used as parameters to be estimated, and the principle of particle filtering in data assimilation is used to realize the model update according to the addition of data. This approach is expected to fit EMR data better while improving the accuracy of risk assessments. The results showed that the accuracy, recall, and F1 value of the improved model were enhanced, and the decision curve analysis showed that the risk assessment model had better clinical utility 23. Therefore, it innovatively solves the data drift problem, in which model accuracy naturally decreases with time without human intervention.

The remainder of this paper is organized as follows. In Section 2, we describe the process of building a model of high-risk patient characteristics and focus on the process of parameter estimation using particle filtering. Section 3 shows the model calculation results for the same test set and compares the model improvement effects before and after the model, and section 4 summarizes the entire article and discusses the issues that require further discussion.

## Construction and content

### Parameter estimation direct filtering algorithm

#### Algorithm proposed

In the study of the NSCLC risk assessment model, most models have low utilization for new patient data. Based on logistic regression, this study attempts to improve the overall effect of the model using regression coefficients and intercepts as parameters for parameter estimation.

The idea of updating parameters by continuously introducing new data is similar to the principle of particle filtering in data assimilation [15]; therefore, according to the logistic regression risk assessment model, this study proposes a parameter estimation method based on the particle filtering process, the parameter estimation direct filtering algorithm. The method regards the parameter process as the only target state in the filtering problem, constructs a filtering algorithm that only inferentially estimates the parameters themselves, and transforms the measurement equation into a combination of the original observation function and system model function to form an integrated observation function. The parameters are updated with the measured values until a steady state is attained, and the final parameters are substituted into a logistic regression relation to evaluate the model results after parameter estimation.

#### Design ideas

Particle filtering includes two processes as follows: prediction and updating processes. The state prediction value at the time k + 1 is obtained from the state equation x_k + 1_ = f(x_k_) + v_k_; the observed value at time k is obtained according to the measurement equation y_k + 1_ = h(x_k + 1_) + w_k + 1_. Subsequently, the state prediction is updated with error compensation to obtain the optimal estimate at time k. Particle filtering parameter estimation typically starts with the augmentation of the state, combining the parameters that are to be estimated with the state equations as a combined augmentation process, which is used as the target state of the filtering process for prediction update [16]. This common approach considers the parameters a constant process and tends to cause filter degradation [16].

The parameter estimation direct filtering algorithm in this study is an inferential estimation of the parameter itself, taking parameter θ as the only state to be estimated. When the state equation is a very high-dimensional model, and parameter θ is a relatively low-dimensional vector, the number of dimensions in the direct filtering algorithm is the same as the parameter θ dimension, which can be regarded as a dimensionality reduction strategy to solve the problem of dimensional catastrophe in the parameter estimation of incremental filtering. Transformation of the equation of state in particle filtering to a zero-dynamic sequence

1 $${\uptheta}_{\textrm{k}+1}={\uptheta}_{\textrm{k}}+{\epsilon}_{\textrm{k}},{\epsilon}_{\textrm{k}}\sim \textrm{N}\left(0,\textrm{Q}\right)$$

where ϵ_k_ is the artificial dynamic noise, and θ_0_ is the initial value of the parameters in the state model. The parameter prediction process {θ_k_}, k ≥ 0 is an artificially defined pseudomorphic process and the patient prognosis data {Y_k_} as a measurement process cannot provide a direct measurement of θ_k_. To make Y_k_ effectively connected to θ introduce variable X_k + 1_, the observation function is defined as

2 $${\textrm{Y}}_{\textrm{k}+1}=\textrm{f}\left({\textrm{X}}_{\textrm{k}},{\uptheta}_{\textrm{k}+1}\right)+{\upxi}_{\textrm{k}+1},{\upxi}_{\textrm{k}+1}\sim \textrm{N}\left(0,R\right)$$

where ξ_k + 1_ is the artificial dynamic noise, θ_k + 1_ is a parameter, X_k_ is a variable value, and Y_k + 1_ is a measured value. The parameter estimate at the moment k is $\textrm{p}\left({\theta}_k\mid\hat{Y}_{1:k}\right)$.

#### Algorithm derivation process

Suppose there are M particles ${\left\{{\zeta}_k^{(m)}\right\}}_{m=1}^M$ at the moment k, the prediction of the direct filtering of the parameter estimation is essentially the addition of artificial noise to the set of particles to obtain the set of predicted particles, i.e. ${\overset{\sim }{\zeta}}_{k+1}^{(m)}={\zeta}_k^{(m)}+{\epsilon}_k^{(m)}$. In turn, the approximate distribution $\overset{\sim }{\uppi}\left({\theta}_k\mid\hat{Y}_{1:k}\right)$ of the target distribution is obtained from the Monte Carlo algorithm [17] as

3 $$\overset{\sim }{\uppi}\left({\theta}_k\mid\hat{Y}_{1:k}\right):= \frac{1}{M}\sum\nolimits_{m=1}^M{\delta}_{{\overset{\sim }{\zeta}}_{k+1}^{(m)}}\left({\theta}_k\right)$$

The Monte Carlo approximation of the posterior distribution is obtained from the Bayesian formula.

4 $$\overset{\sim }{\uppi}\left({\theta}_{k+1}\mid\hat{Y}_{1:k+1}\right):= \frac{\sum_{m=1}^Mp\left(\hat{Y}_{k+1}\mid{\overset{\sim }{\zeta}}_{k+1}^{(m)}\right){\delta}_{{\overset{\sim }{\zeta}}_{k+1}^{(m)}}\left({\theta}_{k+1}\right)}{\sum_{m=1}^Mp\left({\theta}_{k+1}\mid{\overset{\sim }{\zeta}}_{k+1}^{(m)}\right)}$$

where $\overset{\sim }{\uppi}\left({\theta}_{k+1}\mid{\hat{Y}}_{1:k+1}\right)$ is the weighted posterior distribution, approximating the posterior distribution $\textrm{p}\left({\theta}_{k+1}\mid\hat{Y}_{1:k+1}\right)$. $p\left(\hat{Y}_{k+1}\mid{\overset{\sim }{\zeta}}_{k+1}^{(m)}\right)$ is the likelihood function, and $\left({\hat{Y}}_{k+1}\mid{\overset{\sim }{\zeta}}_{k+1}^{(m)}\right):= \exp \left(-\frac{1}{2R}{\left({\textrm{Y}}_{\textrm{k}+1}-\hat{Y}_{k+1}\right)}^2\right)$, to solve the simplex problem, the particles are resampled using a random resampling method.

**Fig. 1:**
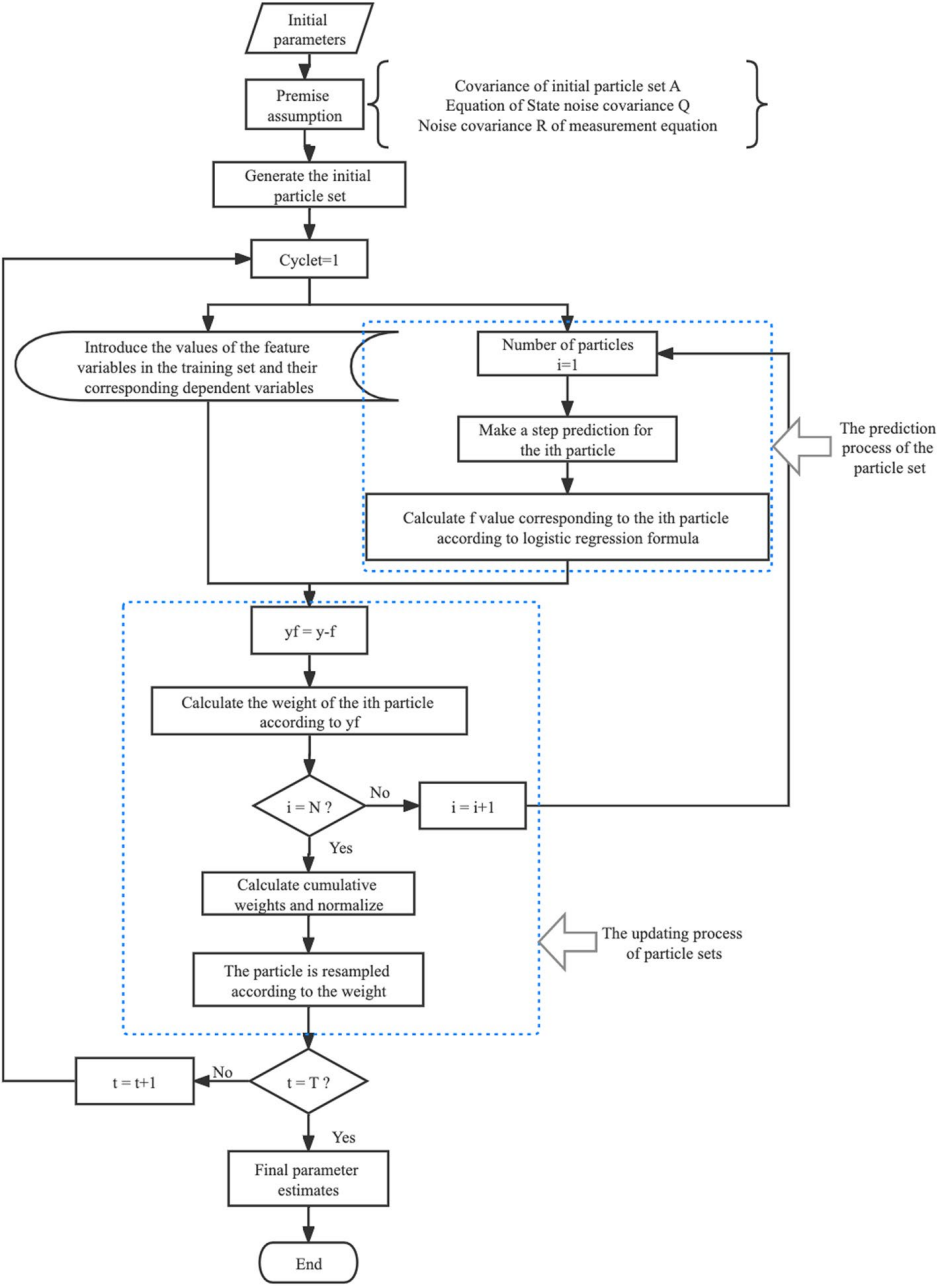
The flowchart of the direct filtering algorithm for parameter estimation

Each prediction and update will yield an approximate probability distribution $\overset{\sim }{\textrm{p}}\left({\theta}_k\mid{\hat{Y}}_{1:k}\right)$, and the corresponding conditional expectation $\overset{\sim }{\textrm{E}}\left({\theta}_k\mid\hat{Y}_{1:k}\right)$ is calculated as the parameter estimate at the moment k, i.e. ${\overset{\sim }{\theta}}_k:= \overset{\sim }{\textrm{E}}\left({\theta}_k\mid\hat{Y}_{1:k}\right)$. Because of the artificial parameter noise *ϵ*_*k*_, the observation noise *ϵ*_*k*_ is a covariance-invariant Gaussian noise in the direct filtering method. To balance the effect of noise, $\overset{\sim }{\theta}:= \frac{1}{nl}\sum_{i=\textrm{l}}^n\overset{\sim }{\textrm{E}}\left({\theta}_k\mid\hat{Y}_{1:k}\right)$ is used as an estimate of parameter θ in practice, and l is a user-defined update step. Figure 1 shows a flowchart of the particle filter for the parameter estimation algorithm.

**Figure:**
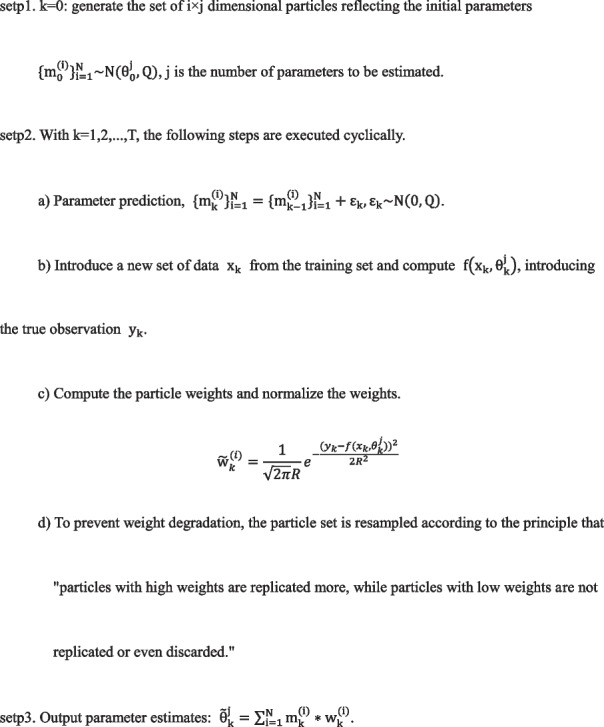
** Algorithm 1** Particle filter parameter estimation

### Empirical analysis

#### Data sources

Clinical data of patients who underwent surgical resection for primary lung cancer at Shanghai Chest Hospital between 2008 and 2018 were collected and organized. According to the 8th edition of TNM staging and combination criteria, patients with distant metastases after preoperative imaging, emergency surgery, preoperative adjuvant radiotherapy, and a history of other malignancies were excluded, and patients with lung cancer postoperative pathological staging of stage I or II were selected, and 1288 patients data were collected. The independent variables were the clinical data of patients, classified into 16 categories with 70 characteristics, of which follow-up data were based on the latest information, and the dependent variables were the overall survival between the date of patient surgery and the time of death.

### Feature dimensionality reduction

To prevent overfitting in the modeling process, it was necessary to reasonably downscale high-dimensional data without losing much possible data information. Through dimensionality reduction, the spatial complexity of the data can be reduced, and the established model has stronger robustness in small datasets. Two problems were solved before feature dimensionality reduction as follows: (1) Features with low variance indicated that this feature explains less of the dependent variable y and contains less available information, so they are deleted. (2) The absolute value of the correlation coefficient of the two features was between 0.6 and 1, indicating that the information carried by these two features was highly similar, and too many similar features will reduce the performance of the algorithm; thus, one of them was retained by setting a threshold. After the feature-expression differentiation process, 60 feature values remained.

#### LASSO regression feature dimensionality reduction

The least absolute shrinkage and selection operator (LASSO) regression is based on linear regression with an L1 regular penalty term. It allows some characteristic indicators with relatively small parameters to regularize directly to zero [18] to achieve dimensionality reduction and obtain characteristic indicators with a high correlation with the survival of patients with lung cancer. As demonstrated, LASSO regression is used to select variables and adjust the complexity of the model when fitting a generalized linear model and is therefore applicable to binary or multivariate, continuous, or discrete variables [11].

**Fig. 2:**
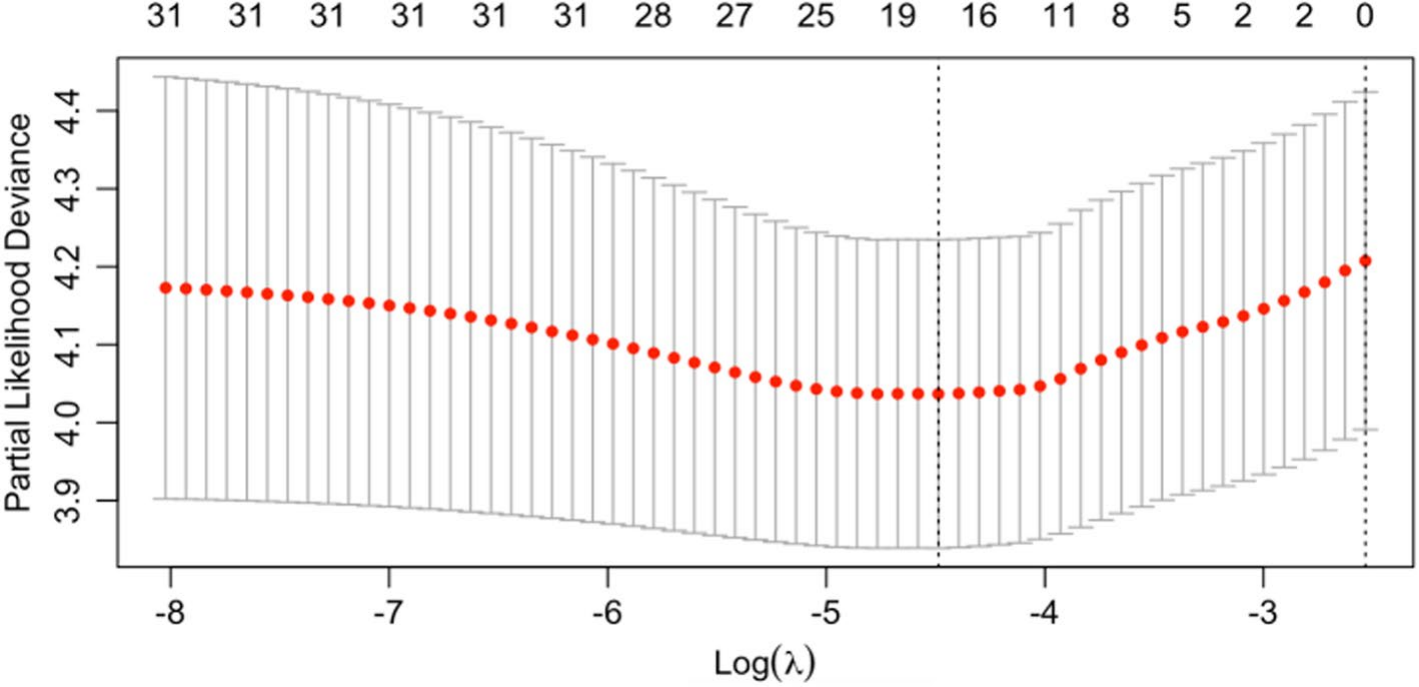
The selection of penalty parameter λ over the graph

The L1 canonical term penalty parameter λ was used for 10-fold cross-validation. Figure 2 shows the selection process of the penalty parameter λ, and the most appropriate penalty parameter λ = 0.0002354. Additionally, using LASSO regression, 33 eigenvalues associated with the degree of survival of patients with lung cancer were identified, including sex, age at onset, number of hospitalizations, total stage, cough and chest pain presence, family history of lung cancer, history of hypertension, if undergone targeted therapy radiotherapy, presence of pericardial effusion, and histological typing, among others.

#### Random Forest feature screening

**Fig. 3:**
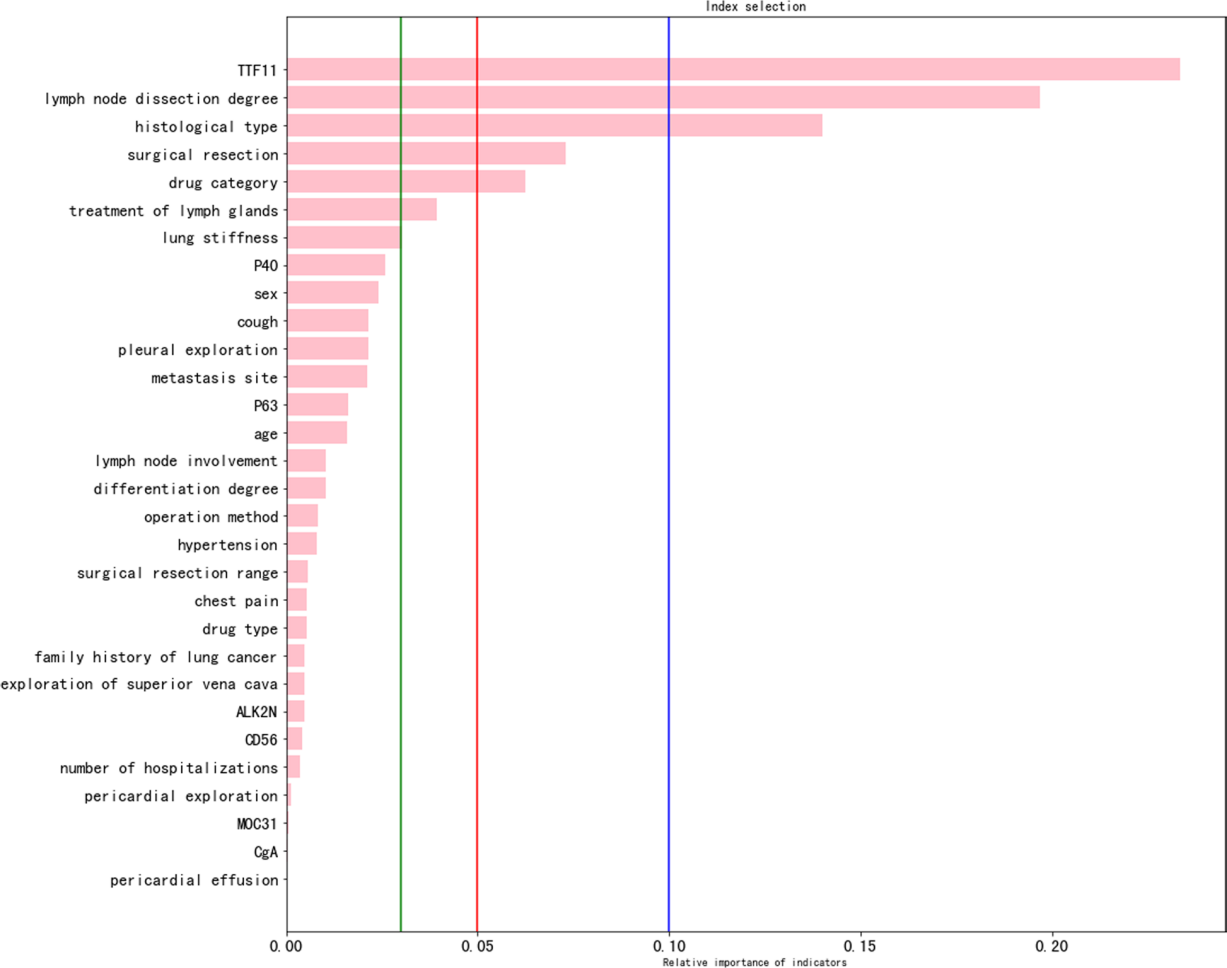
Random forest importance ranking chart

The importance measure of the random forest algorithm can be used as a feature selection tool for high-dimensional data, ranked in descending order of importance to the dependent variable, and variable screening is achieved by setting a threshold [19]. By converting multi-categorical variables to dummy variables among the 33 feature variables, the number of variables will be increased substantially, and the building model will remain relatively highly dimensional. The purpose of feature variable screening using random forest is to identify feature variables that are strongly correlated with the dependent variable and can adequately predict the outcome of the dependent variable with a smaller number of feature variables. General screening is divided into two steps as follows: (1) initial estimation of importance and degree of explanation and sorting in descending order based on both, and (2) determination of weight thresholds and deletion ratios. Thirty-three feature indicators were ranked by random forest importance, as shown in Fig. 3. Taking the modeling dimensions into account and combining it with the literature review, a weight threshold of 0.05 was selected to enter five feature variables into the prognostic survival model of patients with lung cancer. The five variables were pathological thyroid transcription factor-1 (TTF1), degree of lymph node clearance, histological staging, surgical resection, and surgical approach.

#### Model building

The multi-categorical independent variables were converted into dummy variables, and the 3-fold cross-validation method was used to establish a patient survival model with a coefficient significance test. The results showed that the four indicators entered the model, including TTF-1 results in pathology, histological typing, lymph nodes clearing intraoperatively, and the usage of chemotherapeutic hemostatic drugs in the medication record. The output parameters and regression accuracy for the same training set differed according to the amount of data. As the number of samples in the training set increased, the coefficients and intercepts of the model did not change in single digits when increased by approximately 250 entries. Moreover, the overall prediction accuracy of the model was maintained at 90.93%, indicating that the coefficients and intercepts tended to be stable, and the model utilized less data on the characteristics of subsequent patients. The stabilized logistic regression relationship equation is as follows:

$$\ln \frac{\textrm{y}}{1-\textrm{y}}=2.79\ast {\textrm{x}}_1-2.39\ast {\textrm{x}}_2+2.4\ast {\textrm{x}}_3+1.3\ast {\textrm{x}}_4-0.9$$

When applying the parameter estimation direct filtering algorithm to actual data, first, the data from the test set needs to be divided into two parts, including using logistic regression to generate the initial parameters and data continuously introduced in the particle filtering process. Additionally, before the parameter estimation is directly filtered, it should have some premise, including the state of the space model of disturbance and measurement of noise covariance matrix Q, equation of disturbance of the noise covariance R, the initial particle sets the covariance of A, the number of filters N, and cycle time K. Although these variables are custom settings, they must have a certain standard. The shape of the perturbed noise covariance matrix Q is determined according to the number of parameters; here, the coefficient plus intercept has five parameters, and Q is a 5X5 symmetric matrix. The settings of Q, R, and A, among others, can be divided into various experimental schemes, the results of the comparison, and the final selection of the best set of experimental results. Table 1 lists the settings of the experimental scheme and experimental results.

**Table 1:** Different experimental protocols and results

	The first experiment	The second experiment	The third experiment	The fourth experiment
A	N(0,1)	N(0,1)	N(0,1)	N(0,1)
Q	No standardization and no difference	Differentiation	Differentiation and standardization	Differentiation and standardization
R	0.1	0.1	0.1	0.05
N	100	100	100	100
T	608	608	608	608
Accuracy	0.4895	0.7366	0.8556	0.8324
Stability of parameters	poor	poor	poor	poor
	The fifth experiment	The sixth experiment	The seventh experiment	The eighth experiment
A	N(0,1) *0.05	N(0,1)	N(0,1) *0.1	N(0,1)
Q	Differentiation and standardization	Differentiation and standardization *0.05	Differentiation and standardization	Differentiation and standardization *0.1
R	0.1	0.1	0.1	0.1
N	100	100	100	100
T	608	608	608	608
Accuracy	0.85	0.8625	0.8601	0.9184
Stability of parameters	poorer	good	poorer	very good

After many experiments were adjusted, the perturbation noise covariance Q in the state space model was set as a matrix with differentiation with normalization, i.e., the value on the diagonal = corresponding initial parameter value/sum of all initial parameter values, and the value on the non-diagonal = correlation coefficient of the corresponding two feature indicators/sum of all correlation coefficients. The overall Q was reduced by a factor of 10 to ensure that the fluctuations of the particles during the prediction update were not too large and that the parameters could reach a steady state. A smaller set of the perturbation noise covariance R in the measurement equation indicated a greater belief in the measurement data, and a larger R showed a bias toward the initial parameter information. Here, a greater belief in the data is chosen, and *R* = 0.1. Table 2 summarizes the other settings.

**Table 2:** Summary of parameter estimation premise settings

Data Segmentation	Divided into 30% discount	the training dataset 2/3, the test dataset 1/3
Data set Description	Logistic regression	The entire training set, with 858 data
	Particle filtering initial parameters	The first 250 data in the training set
	Filtering process	The remaining 608 data in the training set
Other parameters description	A variance of the initial particle set A	A ~ N(0,1)
	Initial particle set P	P ~ N(initial parameters, A)
	State equation noise covariance Q	A matrix of differentiation and standardized
	Measurement equation noise covariance R	0.1
	Number of particles	100
	Cycle Time K	608

**Fig. 4:**
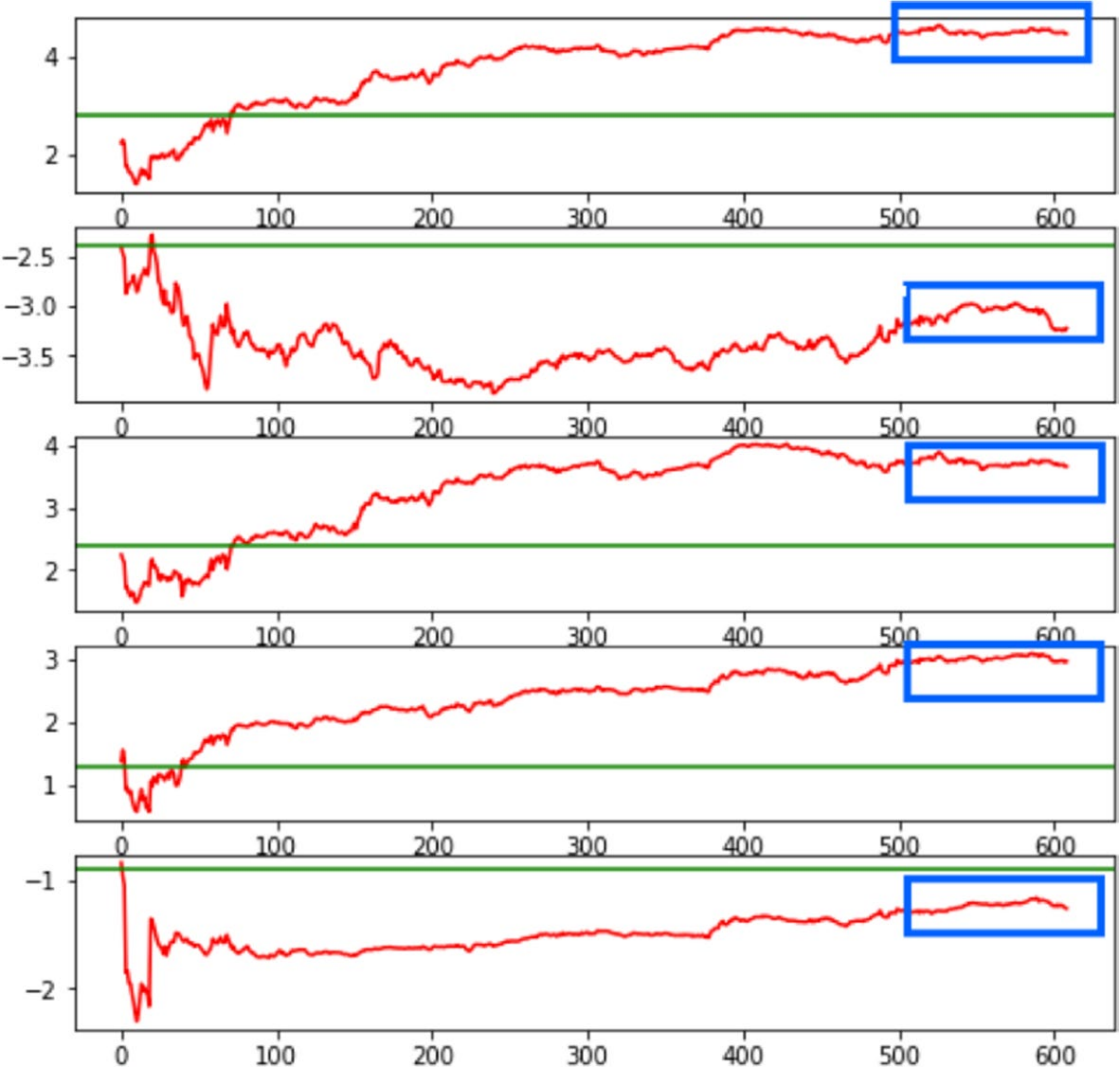
Parameter fluctuation diagram of the direct filtering algorithm for parameter estimation

Figure 4 shows the fluctuations in the parameters during cycling. The green line indicates the parameter results of the overall logistic regression, and the red curve indicates the fluctuation of the parameter estimates after each particle filter prediction update for a total of 608 iterations. From the fluctuation of the parameters, the parameter estimates were basically stable during the last 100 iterations of the particle filter, with the up and down fluctuations not exceeding approximately 0.5, indicating that the customized state equation covariance Q is set reasonably. This result indicated that the true value of the parameter estimates considered by the particle filter is reached, proving the effectiveness of the direct filtering algorithm for parameter estimation.

## Utility and discussion

### Comparison of the results of the same test set

Since the dependent variable of patient survival is unbalanced data, observing only the metric of prediction accuracy is not feasible. Rather, more attention is required on the magnitude of the recall rate of deceased patients in medical prognosis scenarios, i.e., the proportion of patients predicted to die to those who did die. The area under receiver operating characteristics (ROC) curve (AUC) value indicated the effect of the classifier, and the larger the AUC value, the better the classification effect. The same test set was used to measure the overall effect of the model in terms of AUC values, model prediction accuracy, recall rates for surviving patients and deceased patients, and the F1 values to validate the efficacy of the direct filtering algorithm for parameter estimation.

The AUC value of the general risk assessment model based on logistic regression was 0.8, and the overall prediction accuracy was 0.90. Since the number of surviving and deceased patients in the dependent variable data was uneven, the precision and recall F1 values between both surviving and deceased patients needed to be observed. Table 3 shows the results of the model evaluations. The prediction was better for survival patients with a precision rate and recall rate of 0.9, while for death patients, the recall rate was lower than 0.6.

**Table 3:** Overall prediction evaluation index of logistic regression model

Logistic regression accuracy		0.90	
	precision	recall	f1-score
Survival	0.90	0.99	0.9
Death	0.91	0.60	0.73

The parameter estimates of the direct filtering algorithm were applied to the test set, and the model AUC value was 0.84, with a prediction accuracy of 92.09%, which was further improved compared with the direct use of logistic regression, indicating the effectiveness of the direct filtering algorithm for parameter estimation. Table 4 shows the model evaluation results. The precision rate of surviving patients increased by 0.03, and the recall rate decreased by 0.02; however, the F1 value of both could be increased by 0.05, the precision rate of dead patients decreased by 0.04, the recall rate increased by 0.11, and the F1 value increased by 0.05. Therefore, the parameter estimates after particle filtering improved the model precision for surviving or dead patients, especially for the recall rate of the deceased patients. Additionally, the increased recall rate can enable more patients to intervene earlier in all aspects and reduce mortality.

**Table 4:** Logistic regression results after parameter estimation

Logistic regression accuracy		0.9209302325581395	
	precision	recall	f1-score
Survival	0.93	0.97	0.95
Death	0.87	0.71	0.78

## Discussion

The high mortality rate of patients with lung cancer is not only because it is not easily detected in its early stages but also because of its poor prognosis, and some patients experience recurrence after treatment. Therefore, it is important to conduct a prognostic analysis of patients with lung cancer. Furthermore, many types of patient information are stored in EMR [20]. Risk characteristics that are closely related to patient survival and poor prognosis can be obtained by analyzing a large amount of data on patient characteristics. Patients can then be treated differentially according to their risk characteristics to improve their overall survival.

### Effectiveness of direct filtering risk assessment model to avoid model attenuation

In clinical practice, the doctor’s postoperative treatment plan is based on a combination of factors, including the patient’s clinical information and relatively real-time clinical auxiliary test results and the final treatment plan made at that time. Therefore, establishing an adaptive multifactorial clinical risk assessment model has important clinical implications for physician consultation and treatment decisions [21]. In this study, 70 feature indicators, including basic patient information, visit characteristic labels, and clinicopathological factors were analyzed using feature differentiation processing, LASSO regression, and random forest feature selection. The NSCLC multifactor prognostic model was established using the reduced dimensional data, and the adaptive model was realized by the particle filtering direct parameter estimation method. The results showed that the accuracy, recall, and F1 value of the improved model were enhanced, and the decision curve analysis showed that the risk assessment model had better clinical utility [22]. Therefore, it innovatively solves the data drift problem, in which model accuracy naturally decreases with time without human intervention.

### Effectiveness of direct filtering risk assessment model to improve model performance

The NSCLC prognostic model needs to be evaluated for its effectiveness, and the repeatability and generalization ability of the model should be examined. Therefore, a valid model evaluation generally requires validation of its efficacy through a non-training set. In this study, we considered the coefficients and intercepts of the prognostic model as state variables and then used the particle filtering algorithm to implement the optimization of the parameters of the risk assessment model by continuously adding data. The accuracy of the risk assessment model can be improved using this approach. Therefore, we compared the initial and improved models using the same test set. The improved model achieved an accuracy rate of 92.09%, a recall rate of 97% for surviving patients, and a recall rate of 71% for deceased patients. Compared with the initial risk assessment model, the improved model not only has an improved overall accuracy rate but also has a greater increase in the recall rate for surviving and deceased patients.

### Rationality of direct filtering risk assessment model to guide clinical practice

Among the five factors that eventually entered the model, the pathological index of TTF-1, degree of lymph node clearance, and histological typing ranked the top three in the random forest weighting. TTF-1 is an isoform of the thyroid transcription factor. The probability of positive epidermal growth factor receptor (EGFR) mutation is increased in patients with TTF-1-positivity in many foreign and domestic clinical research, and the patients with NSCLC also have a better prognosis [23]. These factors correspond to the positive coefficient in the model. Studies have shown that the degree of lymphatic clearance is an important factor in the surgical treatment of lung cancer [24]. The coefficient of lymph node clearance degree, unscavenged in the risk assessment model, is negative, indicating that the probability of survival is low for patients without lymph node dissection. In this study, the histological classification of lung cancer was adenocarcinoma and non-adenocarcinoma. Studies have shown that women with NSCLC adenocarcinoma have a lower risk of breast cancer than men and that NSCLC adenocarcinoma expresses more TTF-1 [25]. These two factors determined a better prognosis for patients with lung adenocarcinoma than those without adenocarcinoma, which also corresponded to the positive coefficient of histological classification in our risk assessment model. Simultaneously, it justified the rationality of our risk assessment model and indicated that the model would provide guidance for clinical practice.

### Shortcomings and prospects

The risk assessment model developed in this study is based on the parameter estimation direct filtering algorithm combined with data mining of clinical characteristics index data of patients with lung cancer. Although some risk factors were obtained and the prognostic model of patients with NSCLC established by these factors achieved relatively good prediction accuracy using the direct filtering algorithm of parameter estimation, there are still some issues that require further investigation. First, the number of surviving patients and the number of dead patients in the sample data of this study were unbalanced, and more samples are needed to verify the accuracy of this model. Second, dimension reduction can be compared with other approaches, and there may be better attempts at modeling algorithm values for current data. Third, although the model results were validated by machine learning analysis, survival analysis, and ROC curves, the lack of clinical trial makes it seem tenuous.

## Data Availability

The data that support the findings of this study are available from Shanghai Chest Hospital, Shanghai Jiao Tong University but restrictions apply to the availability of these data, which were used under license for the current study, and so are not publicly available. Data are however available from the author reasonable request and with permission of Shanghai Chest Hospital, Shanghai Jiao Tong University.

## Abbreviations

LASSO: Lleast absolute shrinkage and selection operator
TTF1: Thyroid transcription factor-1
NSCLC: Non-small cell lung cancer
TNM: Tumor, node, and metastasis
EMR: Electronic medical records
ML: Machine learning
EnKF: Ensemble Kalman Filter
EGFR: Epidermal growth factor receptor
